# Effect of initial pH, different nitrogen sources, and cultivation time on the production of yellow or orange *Monascus purpureus* pigments and the mycotoxin citrinin

**DOI:** 10.1002/fsn3.1197

**Published:** 2019-09-27

**Authors:** Matej Patrovsky, Kristyna Sinovska, Barbora Branska, Petra Patakova

**Affiliations:** ^1^ Department of Biotechnology University of Chemistry and Technology Prague Prague 6 Czech Republic

**Keywords:** citrinin, *Monascus purpureus*, orange pigments, yellow pigments

## Abstract

*Monascus purpureus* was grown in submerged liquid culture using ammonium sulfate, sodium nitrate, and peptone as nitrogen sources while initial medium pH was adjusted to 2.5, 5.5, 6.5, or 8.0. The combined effect of culture pH and nitrogen source on the biosynthesis of yellow (ankaflavin and monascin) and orange (rubropunctatin and monascorubrin) pigments, plus the mycotoxin citrinin, was evaluated chromatographically. Optimum cultivation conditions, that is, initial pH 2.5 and 8.8 g/L peptone as a nitrogen source, resulted in high levels of production of yellow and orange pigments (sum of pigment concentration 1,138 mg/L) and negligible citrinin concentration (2 mg/L).

## 1 INTRODUCTION

The filamentous fungus *Monascus purpureus* is widely known, especially in Asian countries (China, Korea, Japan, Thailand, Indonesia, Taiwan, Philippines), for its use in the preparation of a traditional food called red fermented rice or red yeast rice (Patakova, 2013). Red yeast rice is known under various Asian names, that is, as angkak (Philippinese), hong qu (Chinese), or beni‐koji (Japan), and is mostly associated either with food coloring/flavoring or with beneficial effects on human health, particularly its positive effects on digestion and against cardiovascular diseases (Patakova, Branska, & Patrovsky, 2016). The fungus is capable of producing six main pigments, that is, yellow (monascin, ankaflavin), orange (rubropunctatin, monascorubrin), and red (rubropuctamine, monascorubramine), plus monacolin K (a statin), mycotoxin citrinin, and other compounds (Feng, Shao, & Chen, 2012). The main question regarding safety and usefulness of food colorings using *Monascus* pigments is their potential contamination with the mycotoxin citrinin, which can be produced together with the pigments.

Despite the long history of use of *Monascus* fungus in food and also as a folk medicine in China almost 2000 years ago, the genome of *M. purpureus* was first sequenced in 2015 (Yang et al., 2015). In addition, biosynthesis of the yellow and orange main polyketide pigments described above was only recently elucidated unambigously in a series of experiments with targeted mutants (Chen et al., 2017). The same is true for the mycotoxin citrinin, the synthesis of which was proposed in 2016 (He & Cox, 2016).

Not only had the biosynthesis of polyketide secondary metabolites remained undiscovered for a long time, there are still many significant flaws in most of the otherwise excellent scientific works dealing with *Monascus* pigments. Traditionally, the color of *Monascus* pigment alcohol extracts is evaluated by spectrophotometric analysis, and usually, three values at the absorption maxima of yellow, orange and red pigments, that is, at 410, 470, and 510 nm, respectively, are compared. However, these values do not correspond with actual amounts of individual pigments in crude ethanol extracts. They might reflect the individual pigment concentrations if it was possible to extract pure individual compounds, but this is not the case. In crude pigment extracts of either *Monascus* mycelium or red yeast rice, in submerged or solid‐state cultivations, respectively, all ethanol‐soluble compounds were extracted together with a mixture of pigments. All compounds in the extract may contribute to the resulting absorbance value, and what is probably more significant, the absorption spectra of *Monascus* pigments overlap with one another. In addition, red *Monascus* pigments are not formed biosynthetically but by a chemical reaction with compounds containing primary amino groups, such as amino acids, nucleotides, and others at an appropriate pH. Such reactions can also proceed during extraction (Shi et al., 2015) unless they are prevented by low pH of the solvent. Another issue in many works focused on *Monascus* pigments is the neglect of citrinin determination despite the known fact that pigment hyper‐producing *Monascus* strains often also produce citrinin.

It has been shown (Carels & Shepherd, 1977; Chen & Johns, 1993, 1994; Shi et al., 2015; Wong, Lin, & Koehler, 1981) that in submerged liquid culture, the nitrogen source together with initial pH is significant factor influencing *Monascus* pigment formation. Thus, in this study, whose main aim is to evaluate properly the effect of culture conditions on the biosynthesis of *Monascus* yellow and orange pigments and the mycotoxin citrinin, the same factors were chosen as variables. To prevent a chemical reaction resulting in the formation of red pigments, 70% ethanol solution acidified to pH 2 was used as a solvent, and to ensure precise analysis of pigments and citrinin, HPLC determination of secondary metabolites was employed. In addition, attention was focused on the intracellular formation of yellow and orange pigments, for which it has been confirmed recently (Chen et al., 2017; Chen, Feng, Molnár, & Chen, 2019) that they are of biosynthetic origin while previously it was assumed that yellow pigments were formed chemically from orange ones.

## 2 MATERIALS AND METHODS

### 2.1 Microorganism

*Monascus purpureus* DBM 4360, isolated from a nonsterile dried red fermented rice sample delivered from China (PRC), was maintained on Sabouraud agar slants at 4°C. Taxonomic classification of the isolated *Monascus* strain was confirmed by sequencing of the gene for 18S rRNA. The strain was deposited in the Department of Biochemistry and Microbiology (DBM), University of Chemistry and Technology Prague.

### 2.2 Culture media

Inoculum culture medium consisted of glucose 20 g, peptone (Roth) 10 g, yeast extract (Merck) 3 g, KCl 0.5 g, KH_2_PO_4_ 4 g, ZnSO_4_.7H_2_O 0.01 g, and FeSO_4_.7H_2_O 0.01 g in 1 L of demineralized water. Culture medium consisted of glucose 40 g, one of the investigated nitrogen sources (NaNO_3_ 6.44 g, (NH_4_)_2_SO_4_ 5.00 g, peptone 8.84 g), KCl 0.5 g, KH_2_PO_4_ 4 g, ZnSO_4_.7H_2_O 0.01 g, MgSO_4_.7H_2_O 0.5 g, and FeSO_4_.7H_2_O 0.01 g in 1 L of demineralized water. The pH of the inoculum was adjusted to selected values (2.5, 5.5, 6.5, 8.0) using 1M HCl or 1M NaOH before sterilization of the medium. Salts, HCl, and NaOH for media preparation were purchased from Penta, Czech Republic.

### 2.3 Cultivation

For inoculum cultivation, a suspension of spores was collected by washing the slant with sterile water and 2 ml of spore suspension was inoculated into 50 ml of inoculum culture medium in a 250 ml Erlenmeyer flask. This cultivation was carried out at 30°C, 100 rpm for 30 hr in a shaker (New Brunswick—Scientific).

An aliquot of inoculum culture (5 ml) was then inoculated into 50 ml of culture medium in a 250 ml Erlenmeyer flask and incubated at 30°C, 100 rpm for 10 days (in the dark). The dried biomass concentration of the inoculum culture was 1.5 ± 0.2 g/L.

For cultivation time test: NaNO_3_ was used as a nitrogen source. The cultivation was carried out for 6, 10, 14, 20, 24 days at 30°C at 100 rpm (in the dark). The initial pH was set at 5.5.

All cultivations were carried out in triplicate, and the results are reported as the average of these values.

### 2.4 Extraction

After cultivation, the fermented medium was filtered through Whatman 1 filter paper. The separated fungal mycelium was quantitatively transferred to a 250 ml Erlenmeyer flask, and 20 ml of 70% ethanol about pH 2 (pH was adjusted by HCl 1M) was added to the flask. Extraction was performed at 30°C, 100 rpm for 1 hr using a shaker (New Brunswick—Scientific).

After extraction, the solvent was filtered through filter paper Whatman 1. The extract was then used for the determination of intracellular pigments and citrinin by HPLC.

### 2.5 HPLC analysis

HPLC (Agilent 1100 system) was used to determine the six main *Monascus* pigments and citrinin using the following conditions: Watrex SN7632 column, 250x4 mm, Reprosil 100 C18 packing (mesh 5 µm); the mobile phase: 360 ml demineralized water, 640 ml acetonitrile, and 0.5 ml of H_3_PO_4_; and the flow rate 1 ml/min. For the determination of yellow and orange pigments, the photodiode detector at 390 nm for yellow and 470 nm for orange pigments was used. For the determination of mycotoxin citrinin, the fluorescence detector having an emission at 331 nm and excitation at 500 nm was used.

The standards of mycotoxin citrinin (Sigma‐Aldrich), yellow pigment monascin (Sigma‐Aldrich), and orange pigment rubropuctatin (1717 CheMall Corporation) were used as reference samples. The pigment ankaflavin is not easily commercially available; thus, the quantification of ankaflavin was carried out from the calibration curve of monascin. We assumed that chromogenic cores of both monascin and ankaflavin are the same and both compounds vary only in the length of one substituent; therefore, we expected a similar extinction coefficient for both pigments. The final concentration of ankaflavin, obtained from the calibration curve of monascin, was multiplied by the factor 1.079 to consider the difference in molar masses of monascin and ankaflavin, and its amount was expressed as monascin equivalent. Similarly, the quantification of monascorubrin was carried out from the calibration curve of rubropunctatin and the final concentration of monascorubrin was multiplied by the factor 1.078 and its amount was expressed as rubropunctatin equivalent.

For the confirmation of identity and purity of the individual pigments, MS‐HPLC (Orbitrap LC‐MS; Thermo Fisher Scientific) was performed. Based on knowledge of mass spectra of particular pigments, we detected ions m/z 354 (rubropunctamine), m/z 382 (monascorubramine), m/z 355 (rubropunctatin), m/z monascorubrin, m/z 359 (monascin), and m/z 387 (ankaflavin) and confirmed the identity of individual compounds, see Figure S1.

## 3 RESULTS AND DISCUSSION

Based on microscopic observations, the isolated strain *M. purpureus* DBM 4,360 preferred sexual reproduction, that is, formation of ascospores to asexual reproduction, that is, conidia formation, and produced different amounts of pigments and citrinin, but not monakolin K, independent of culture conditions. In our work, we focused on determining yellow and orange pigments together with citrinin by chromatographic analysis. The red pigments are made by the reaction of orange pigments with compounds containing an amino group, and the reaction is followed by the formation of a mixture of red pigments, which were not sufficiently separated by HPLC. For this reason, the red pigments were not analyzed. Manipulation of pigment samples was handled carefully to prevent chemical compositional changes in pigment extracts, that is, the extraction was performed at pH 2.0 and the extracts were kept in the dark.

### 3.1 Influence of initial pH and nitrogen source on the production of *Monascus* pigments and citrinin during submerged liquid cultivation in shaken flasks

**Figure 1:**
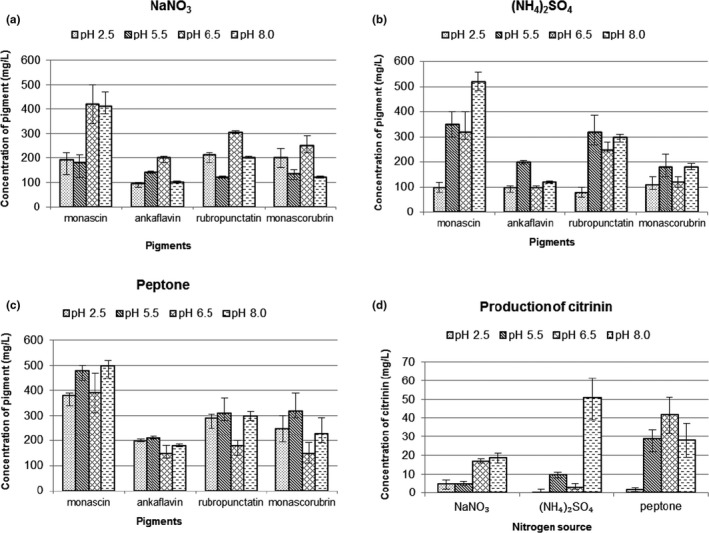
Production of *Monascus* pigments or citrinin with different nitrogen sources at different initial pH; a, b, c—production of yellow and orange pigments, d‐citrinin production. Culture medium composition: glucose 40 g, nitrogen source NaNO_3_ 6.44 g/ (NH_4_)_2_SO_4_ 5.00 g/peptone 8.84 g, KCl 0.5 g, KH_2_PO_4_ 4 g, ZnSO_4_ 0.7H_2_O 0.01 g, MgSO_4_.7H_2_O 0.5 g, and FeSO_4_.7H_2_O 0.01 g in 1 L of demineralized water, initial pH was adjusted to 2.5, 5.5, 6.5, and 8.0. The cultivation conditions: 30°C, 100 rpm, 10 days (in dark). Ankaflavin and monascorubrin concentrations were expressed as monascin/rubropunctatin equivalents

In the first part of our experimental work, the influence of nitrogen source and initial pH of the cultivation medium on the production of yellow and orange *Monascus* pigments and the mycotoxin citrinin were studied at the same time. Nitrogen sources, ammonium sulfate, sodium nitrate, and peptone, were used in different amounts in order to achieve equal concentrations of nitrogen (1 g/L) in the medium. Pigment concentrations in the ethanol extracts ranged from 100 to 500 mg/L, see Figure 1a,b,c, while citrinin concentrations varied from 1 to 50 mg/L (Figure 1d). Fungal growth, expressed as the concentration of dry mycelium weight under the described conditions, and final pH are shown in Table 1.

**Table 1:** Biomass concentration and final pH using different nitrogen sources and different initial pH values

NaNO_3_		DW (g/L)	(NH_4_)_2_SO_4_		DW (g/L)	Peptone		DW (g/L)
Initial pH	Final pH		Initial pH	Final pH		Initial pH	Final pH	
2.5	6.6 ± 0.3	6.9 ± 0.7	2.5	2.9 ± 0.1	12.6 ± 1.5	2.5	2.6 ± 0.1	8.1 ± 0.9
5.5	6.6 ± 0.4	8.6 ± 2.7	5.5	2.5 ± 0.1	9.5 ± 0.7	5.5	6.8 ± 0.1	13.5 ± 1.5
6.5	7.1 ± 0.4	7.9 ± 2.1	6.5	2.3 ± 0.1	10.6 ± 0.3	6.5	7.1 ± 0.1	12.6 ± 0.5
8.0	8.0 ± 0.1	9.4 ± 1.4	8.0	3.1 ± 0.1	12.6 ± 2.0	8.0	7.1 ± 0.1	12.9 ± 0.5

Note

Culture medium composition: glucose 40 g, nitrogen source: NaNO_3_ 6.44 g/ (NH_4_)_2_SO_4_ 5.00 g/peptone 8.84 g, KCl 0.5 g, KH_2_PO_4_ 4 g, ZnSO_4_.7H_2_O 0.01 g, MgSO_4_.7H_2_O 0.5 g, and FeSO_4_.7H_2_O 0.01 g in 1 L of demineralized water, initial pH was adjusted to 2.5, 5.5, 6.5, and 8.0. The cultivation conditions: 30°C, 100 rpm, 10 days (in dark).

Abbreviation: DW—dry weight of the mycelium.

From the results in Figure 1 and Table 1, it can be seen that the fungal strain *M. purpureus* DBM1 was able to grow and produce pigments over a wide spectrum of pH values and nitrogen sources. The pH changes within the culture using different nitrogen sources followed expected patterns (see Table 1). If sodium nitrate was used, the resulting pH increased or remained constant compared with the initial pH due to consumption of hydrogen ions during nitrate assimilation (nitrate to nitrite to ammonia reduction) while in the case of ammonium sulfate, assimilation of ammonia was associated with release of H^+^ cations, resulting in acidification of the medium. Such a pH development in *Monascus* submerged cultures was described for the first time by Carels & Shepherd (1977). Regarding fungal growth, use of nitrate as a nitrogen source represented a limiting factor (see Table 1).

Regarding the yield of pigments and minimal citrinin concentration, the best culture conditions included use of peptone as a source of nitrogen combined with an initial pH of 2.5 (see Figure 1c,d, Tables 1 and 2). In this case, concentration of yellow and orange pigments (sum of yellow and orange pigments) in the mycelium extract was 1,138 mg/L, while citrinin concentration was 2 mg/L. Specific production of yellow and orange pigments was high (141.3 mg/g) under these conditions (see Table 2). Nevertheless, the highest specific yellow and orange pigment production, that is, 147.4 mg/g, see Table 2, was reached using NaNO_3_ as the nitrogen source. Using nitrate as a nitrogen source results in limitation of growth but at the same time stimulation of ascospore, conidia, and pigments formation (Wong et al., 1981). In the same time, pH of the culture medium is increased which might promote the formation of red pigments by reaction of orange ones with compounds containing primary amino group, but these are absent in the medium. Therefore, the use of nitrates leads in particular to the production of orange and yellow pigments, and together with the growth limitation, this explains high specific yellow and orange pigment production.

**Table 2:** Comparison of specific pigments production (mg of pigments per g of biomass)

Initial pH	NaNO_3_		(NH_4_)_2_SO_4_		Peptone	
	Yellow pigments	Orange pigments	Yellowpigments	Orange pigments	Yellowpigments	Orange pigments
(mg/g)						
2.5	44.2	58.7	15.9	15.1	72.8	68.5
5.5	38.4	29.1	57.9	52.6	50.7	45.6
6.5	77.2	70.2	39.6	35.8	50.8	27.0
8.0	53.7	34.0	49.6	38.9	53.5	40.7

Note

Specific pigments production was calculated for sum of yellow (monascin and ankaflavin) and orange (rubropunctatin and monascorubrin) pigments. Sum of yellow or orange pigments was divided by dried weight of the mycelium while all values were related to 1l.

It is also interesting that while in the case of yellow pigments, monascin prevailed under all conditions, in the case of orange pigments, rubropuctatin and monascorubrin concentrations did not differ too much. Ankaflavin and monascin differed in the length of side chain on 2`C in the pigment structure (Chen et al., 2017), and these side chains are synthesized by the fatty acid synthase (FAS) complex with a contribution from acetyl‐transferase. However, little is known about the regulation of FAS. In an older study (Chen & Johns, 1994), ankaflavin production was inhibited by using nitrate as a nitrogen source; this was not confirmed in our study. However, in a study performed with *Monascus ruber* (Huang et al., 2017) under very similar culture conditions using ammonium sulfate as a nitrogen source, the concentration of monascin was higher than that of ankaflavin. The pH value of 2.5 was found to be optimal for yellow pigment formation by *Monascus* spp. (Yongsmith, Tabloka, Yongmanitchai, & Bavavoda, 1993). Monascin and ankaflavin exhibit beneficial health effects such as regulation of blood lipids (Lee, Hung, Hsu, & Pan, 2013; Lee, Wen, Hsu, & Pan, 2018) or suppression of Alzheimer's disease risk factors (Lee, Lin, Hsu, & Pan, 2015), and therefore their potential use as food colorants might actually valorize the colored foods.

Citrinin production was effectively suppressed by low pH (2.5) which seems to be an easy and efficient way to cope with the problem of contamination with this mycotoxin, where ethanol extraction of fungal mycelium is carried out after submerged cultivation. Our results thus confirmed published data by Kang, Zhang, Wu, Wang, & Park (2014) that proposed a similar method to reduce citrinin synthesis. The other way is addition of medium chain length (8–12 carbons) fatty acids in small amounts (milligrams per liter) to the cultivation medium (Hajjaj et al., 2000), resulting in the formation of corresponding methylketones. Both fatty acids and methylketones cause proliferation of peroxisomes, resulting in the degradation of citrinin (Hajjaj et al., 2000). The only citrinin limit for foods in the EU is that for *Monascus* red yeast rice (a food supplement) where the maximum citrinin concentration should not exceed 2000 µg/kg (Commission Regulation (EU) No 212/2014), the daily dose in 0.5 g capsules is 1 µg. In our case, a citrinin concentration of 2000 µg was reached in 1 L of ethanol extract under low pH culture conditions. If we assume that milliliters of the *Monascus* pigment extract will be used for food coloring, the maximum daily consumption of citrinin allowed by EU regulation would not be exceeded. The precise mechanism of citrinin toxicity is still unknown (EFSA Panel of Contaminants in the Food Chain (CONTAM), 2012). Citrinin can be produced by some species of *Penicillium, Aspergillus,* and *Monascus*, and its presence in food is especially troublesome if it is present in a mixture with ochratoxin A. Ochratoxin A and citrinin mixtures exhibit an additive effect in cytotoxicity studies (Föllmann, Behm, & Degen, 2014), and problems can also occur with the quantification of both compounds in these mixtures at certain pH values (Bazin et al., 2013). However, the production of ochratoxin A has never been reported by any *Monascus* species and it seems that a pathway for its production is lacking in the known *Monascus* genomes.

### 3.2 Influence of cultivation time on the production of Monascus pigments and citrinin

**Figure 2:**
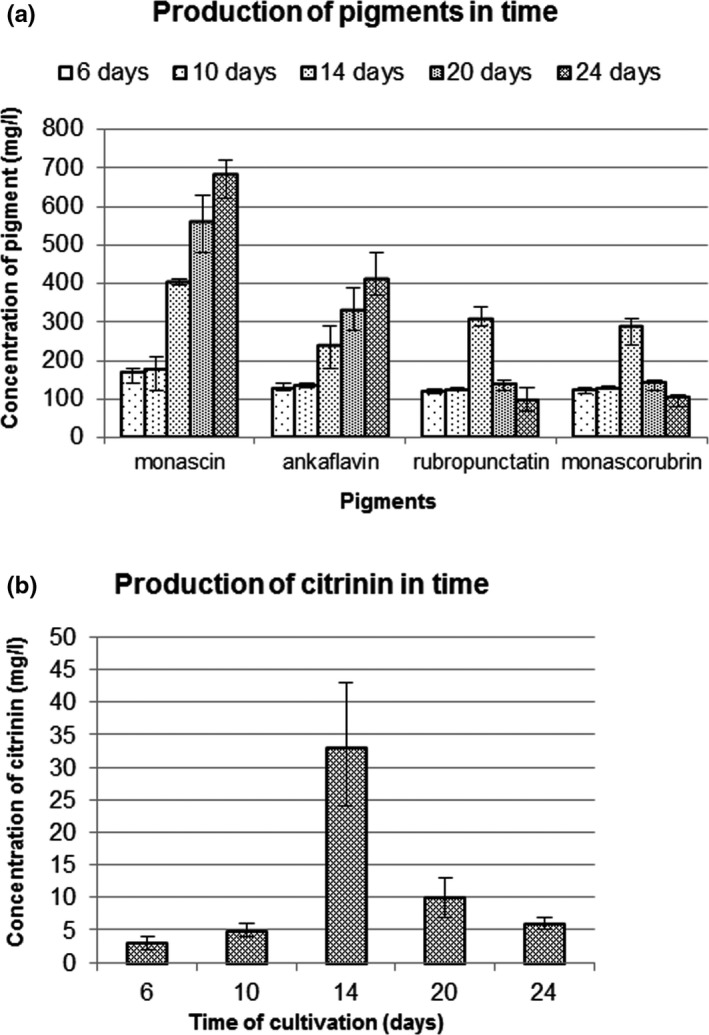
Influence of cultivation time on the production of *Monascus* pigments and citrinin. Culture medium composition: glucose 40 g, nitrogen source NaNO_3_ 6.44 g, KCl 0.5 g, KH_2_PO_4_ 4 g, ZnSO_4_.7H_2_O 0.01 g, MgSO_4_.7H_2_O 0.5 g, and FeSO_4_.7H_2_O 0.01 g in 1 L of demineralized water. The cultivation conditions: 6, 10, 14, 20, and 24 days, 30°C, 100 rpm. The initial pH 5. Ankaflavin and monascorubrin concentrations were expressed as monascin/rubropunctatin equivalents

The fungus grew until day 10 (Table 3) while production of yellow pigments (monascin, ankaflavin) increased until day 24 of cultivation (see Figure 2a). In contrast, the synthesis of orange pigments (rubropunctatin, monascorubrin) occurred only until day 14 of cultivation. After this time, there was a reduction in the level of orange pigments. This may be caused by mycelial cell damage in the later days of cultivation and an easier reaction of amino‐containing substances with orange pigments to form red pigments. Such a reaction was also supported by the convenient pH of the medium (see Table 3). The continuing production of yellow pigments in stationary/death phase of growth corresponds with results obtained for *M. purpureus* in starch medium with nitrate and ammonium salts as nitrogen sources (Lv et al., 2018).

**Table 3:** Time dependence of biomass concentration and final pH

Time (days)	DW (g/L)	final pH value
6	9.0 ± 1.3	6.1 ± 0.5
10	12.6 ± 0.8	6.6 ± 0.4
14	11.6 ± 0.7	7.1 ± 0.1
20	9.8 ± 0.2	7.3 ± 0.3
24	9.7 ± 0.4	7.3 ± 0.2

Note

Culture medium composition: glucose 40 g, nitrogen NaNO_3_ 6.44 g, KCl 0.5 g, KH_2_PO_4_ 4 g, ZnSO_4_.7H_2_O 0.01 g, MgSO_4_.7H_2_O 0.5 g, and FeSO_4_.7H_2_O 0.01 g in 1 L of demineralized water. The cultivation conditions: 6, 10, 14, 20, and 24 days, 30°C, 100 rpm. The initial pH 5.5.

The citrinin concentration increased until day 14 and then decreased (Figure 2b). This is most likely due to degradation of citrinin. It has already been demonstrated that citrinin is degraded to citrinin H2 at a pH above 7.0 (Bazin et al., 2013; Hirota, Mehta, Yoneyama, & Kitabatake, 2002), and in our case, the pH was 7.3 (see Table 3). Citrinin H2, unlike citrinin, has a much lower absorbance at 320 nm compared with citrinin (Hirota et al., 2002) and exhibits significantly lower toxicity (Hirota et al., 2002). Table 3 shows that fungal growth ended by day 10. Both degradation of citrinin and a decrease in orange pigments (see Figure 2) corresponded with probable mycelium lysis (see Table 3). The concentration of citrinin decreased by day 14, in contrast to the finding of Pastrana, Loret, Blanc, & Goma (1996), who described an increase in citrinin concentration after cessation of fungal growth. However, the pH (which was not measured in that study) was probably low due to the use of monosodium glutamate as a nitrogen source. Therefore, it is possible that in our case, citrinin production did not cease at day 14 but that the observed decrease in citrinin was caused by spontaneous citrinin transformation into citrinin H2, as mentioned above.

## 4 CONCLUSION

This is one of the few studies that has evaluated the real biosynthetic production of yellow and orange pigments together with citrinin using HPLC analysis for all compounds using a method of extraction that efficiently prevented chemical transformation of orange to red pigments during extraction. Under our cultivation conditions, at an initial pH of 2.5 and using 8.8 g/L peptone as a nitrogen source, production of citrinin was negligible (about 2 mg/L) while both specific and absolute production of yellow and orange pigments (sum of yellow and orange pigments was 1,138 mg/L) remained high. These conditions might be beneficial in the production of *Monascus* pigments for use in the food industry, where the use of genetically modified strains might be considered problematic due to public aversion to genetically modified organisms.

## CONFLICT OF INTERESTS

The authors declare that they do not have any conflict of interest.

## ETHICAL STATEMENTS

This study does not involve any human or animal testing.
