# Supplementary material for: Effect of initial pH, different nitrogen sources, and cultivation time on the production of yellow or orange Monascus purpureus pigments and the mycotoxin citrinin
Source: Food Sci Nutr. 2019 Sep 27;7(11):3494–500. doi: 10.1002/fsn3.1197 (PMC6848812; doi:10.1002/fsn3.1197)

Supplement 1

**Effect of initial pH, different nitrogen sources and cultivation time on the production of yellow or orange *Monascus purpureus* pigments and the mycotoxin citrinin**

Matej Patrovsky, Kristyna Sinovska, Barbora Branska, Petra Patakova^*^

Department of Biotechnology, University of Chemistry and Technology Prague, Technicka 6, 166 28 Prague 6, Czech Republic

^*^corresponding author, e-mail: petra.patakova@vscht.cz


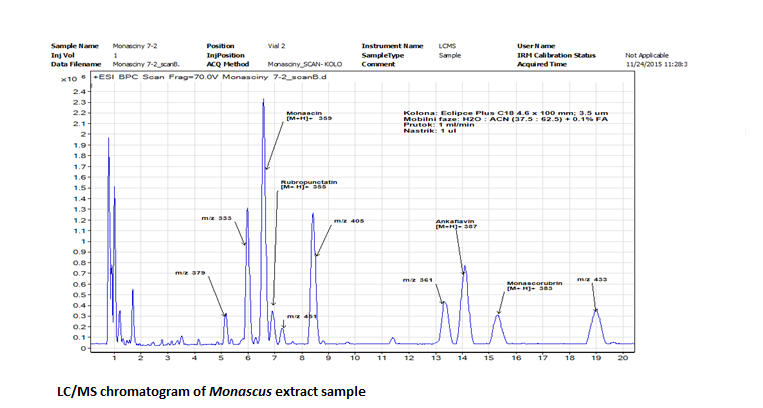

Supplement: Supplementary file 1 [file FSN3-7-3494-s001.docx]
